# Semaglutide Modulates Extracellular Matrix Production of LX-2 Cells via Exosomes and Improves Metabolic Dysfunction-Associated Steatotic Liver Disease (MASLD)

**DOI:** 10.3390/ijms25031493

**Published:** 2024-01-25

**Authors:** Maria Principia Scavo, Giuseppe Lisco, Nicoletta Depalo, Federica Rizzi, Sara Volpe, Valentina Arrè, Livianna Carrieri, Maria Notarnicola, Valentina De Nunzio, Maria Lucia Curri, Giovanni De Pergola, Giuseppina Piazzolla, Gianluigi Giannelli

**Affiliations:** 1 Laboratory of Personalized Medicine, National Institute of Gastroenterology, IRCCS DeBellis, 70013 Castellana Grotte, BA, Italy; valentina.arre@irccsdebellis.it (V.A.); livianna.carrieri@irccsdebellis.it (L.C.); 2 Interdisciplinary Department of Medicine, School of Medicine, University of Bari “Aldo Moro”, Piazza Giulio Cesare 11, 70124 Bari, BA, Italy; giuseppe.lisco@uniba.it (G.L.); svolpe.doc@gmail.com (S.V.); giuseppina.piazzolla@uniba.it (G.P.); 3 Institute for Chemical-Physical Processes, Italian National Research Council (IPCF)-CNR SS Bari, Via Orabona 4, 70125 Bari, BA, Italy; n.depalo@ba.ipcf.cnr.it (N.D.); f.rizzi@ba.ipcf.cnr.it (F.R.); 4 Laboratory of Nutritional Biochemistry, National Institute of Gastroenterology, IRCCS “S. de Bellis” Research Hospital, 70013 Castellana Grotte, BA, Italy; maria.notarnicola@irccsdebellis.it (M.N.); valentina.denunzio@irccsdebellis.it (V.D.N.); 5 Dipartimento di Chimica, Università degli Studi di Bari Aldo Moro, Via Orabona 4, 70125 Bari, BA, Italy; marialucia.curri@uniba.it; 6 Center of Nutrition for the Research and the Care of Obesity and Metabolic Diseases, National Institute of Gastroenterology IRCCS “Saverio de Bellis”, 70013 Castellana Grotte, BA, Italy; giovanni.depergola@irccsdebellis.it; 7 Scientific Direction, National Institute of Gastroenterology IRCCS “De Bellis,” Via Turi 27, 70013 Castellana Grotte, BA, Italy; gianluigi.giannelli@irccsdebellis.it

**Keywords:** metabolic dysfunction-associated steatotic liver disease, MASLD, type 2 diabetes, exosomes, glucagon-like peptide 1 receptor agonist, HEPA-REG, LX-2 cells

## Abstract

Metabolic dysfunction-associated steatotic liver disease (MASLD) is closely related to some metabolic disorders, such as central obesity and type 2 diabetes (T2D). Glucagon-like peptide 1 receptor agonists (GLP-1RAs), such as semaglutide, may have therapeutic roles in MASLD associated with T2D. This study aims to investigate the molecular mechanisms underlying the effectiveness of semaglutide on MASLD in terms of progression from liver steatosis to fibrosis. We characterized exosomes from ten patients with type 2 diabetes (T2D) before (T0) and after 12 months (T12) of treatment with once-weekly subcutaneous semaglutide. Six of ten patients were considered responders to therapy (R) based on MASLD severity downgrading by at least one class according to a validated ultrasonographic (US) score. Normal hepatocytes (HEPA-RG) and stellate (LX-2) cells were challenged with exosomes from R and NR patients, isolated before and after 12 months of therapy. Exosomes from both R and NR patients isolated at T0 significantly affected LX-2 viability. After 12 months of treatment, only those isolated from R patients restored cell viability, whereas those from NR patients did not. No effects were observed on HEPA-RG cells. Exosomes at T12 from R but not from NR patients significantly decreased the production of α-SMA, a marker of LX-2 activation, a liver stellate cell model, and ph-SMAD2 and CTGF, involved in fibrosis processes. TGF-β1 was not modulated by the exosomes of R and NR patients. As a downstream effect, Vimentin, Collagen 1A1, and Fibronectin extracellular matrix components were also downregulated, as measured by droplets digital PCR. In conclusion, these results shed light on the potential effectiveness of semaglutide in improving liver fibrosis in MASLD.

## 1. Introduction

Metabolic dysfunction-associated steatotic liver disease (MASLD) is a metabolic disorder affecting up to a quarter of the world population, characterized by lipid deposition in hepatocytes (hepatic steatosis) and at least one of five cardiometabolic risk factors, i.e., high body mass index (BMI) or waist circumference values, hypertension, hypertriglyceridemia, low high-density lipoprotein (HDL) cholesterol levels, fasting hyperglycemia or diagnosis of diabetes mellitus, and/or ongoing therapy for any of these risk factors [1]. MASLD can evolve into metabolic dysfunction-associated steatohepatitis (MASH), fibrosis, cirrhosis, and hepatocellular carcinoma [2]. Overweight, obesity, metabolic syndrome, and type 2 diabetes (T2D) are the leading risk factors for MASLD [3,4,5].

In patients with T2D, glucagon-like peptide-1 receptor agonists (GLP-1RAs) have been shown to regulate blood glucose while also promoting weight loss [6,7] to provide renal and cardiovascular protection [8,9] and improve body composition [10]. The incretin family is gut-derived hormones rapidly secreted after meal consumption by L and K intestinal cells. Incretins, such as GLP-1, stimulate pancreatic β-cells to release insulin and may suppress glucagon secretion from α-cells. These effects are mediated by GLP-1 binding to islet GLP-1 receptors [11]. 

Subcutaneous semaglutide, a long-acting GLP-1 RA, is one of the most potent molecules of the class approved for T2D, and recently, its potential therapeutic effect on hepatic steatosis has been reported, also because the expression of the GLP-1 receptor in liver parenchyma is still a debated issue [12].

Exosomes are extracellular vesicles, ranging in size from 30 to 150 nm, released in the extracellular space and capable of transferring information from cells to other cells, thus modulating gene expression and, consequently, the cell phenotype [13]. In the evolution of MASLD, the exosomes and their cargo have been reported to play an important role in the degeneration of liver tissue [13]. For these reasons, the study of the composition of exosomal cargo could lead to the identification of new biomarkers for the evaluation of MASLD, which assesses its severity [14,15,16].

This study aims to assess the effect of exosomes derived from patients with type 2 diabetes and MASLD treated with subcutaneous semaglutide on the early formation of fibrotic scaffolding implemented by normal hepatic (HEPA-RG) and stellate (LX2) cells in an extracellular matrix. 

## 2. Results

### 2.1. Patient Characteristics

In total, 48 patients with type 2 diabetes and clinically established MASLD were treated with once-weekly subcutaneous semaglutide for 52 weeks, as described elsewhere [17]. Among them, 10 consecutive patients treated with semaglutide 0.5 mg/week, from whom we obtained venous samples for exosome extraction and analyses, were included in this pivotal study. Patients were classified as responders (Rs) to the effects of semaglutide on the improvement of hepatic steatosis as assessed by ultrasonography (US) if they had a reduction of at least one class of liver steatosis severity after 12 months of treatment with subcutaneous semaglutide, or non-responders (NRs) if otherwise. BMI reduction was considered significant when the change was more than 5% from the initial value. The characteristics of patients before and after semaglutide therapy are described in Table 1. Patient ages ranged from 42 to 71 years. Two of them were diagnosed with type 2 diabetes at T0, while the others had received a diagnosis of T2D 1 to 20 years earlier. All patients were obese according to BMI values calculated at T0 and had a variable level of glucose control (glycated hemoglobin, HbA1c from 34 to 73 mmol/mol). Liver fibrosis scores, namely the aspartate aminotransferase to platelet ratio index (APRI) and the fibrosis-4 index for liver fibrosis (FIB-4), estimated that most of them were at low risk of liver fibrosis (APRI score < 0.5, and FIB-4 < 1.45), with only two patients showing FIB-4 values predicting a moderate level of liver fibrosis (patients number 4 and 8). Hepatic steatosis was diagnosed in all patients using the hepatic steatosis index (HSI > 30), as also confirmed by liver ultrasound (US), which assessed hepatic steatosis as moderate (class 2) in seven patients to severe (class 3) in three.

Table 1 also shows that after 12 months of once-weekly semaglutide therapy, most patients showed a reduction in HbA1c values and body weight, eight patients being considered responders for the latter parameter at T12 (reduction in BMI > 5% from baseline values). Note that patients’ response to semaglutide in terms of BMI reduction or US-grading improvement liver steatosis (reduction of at least one US classification class at T12) did not necessarily coincide in all patients (see column 3 of Table 1). Finally, the two patients exhibiting FIB-4 higher than 1.45 at T0 showed normalized values at T12 (see Table 1).

### 2.2. Characterization of Exosomes 

**Figure 1:**
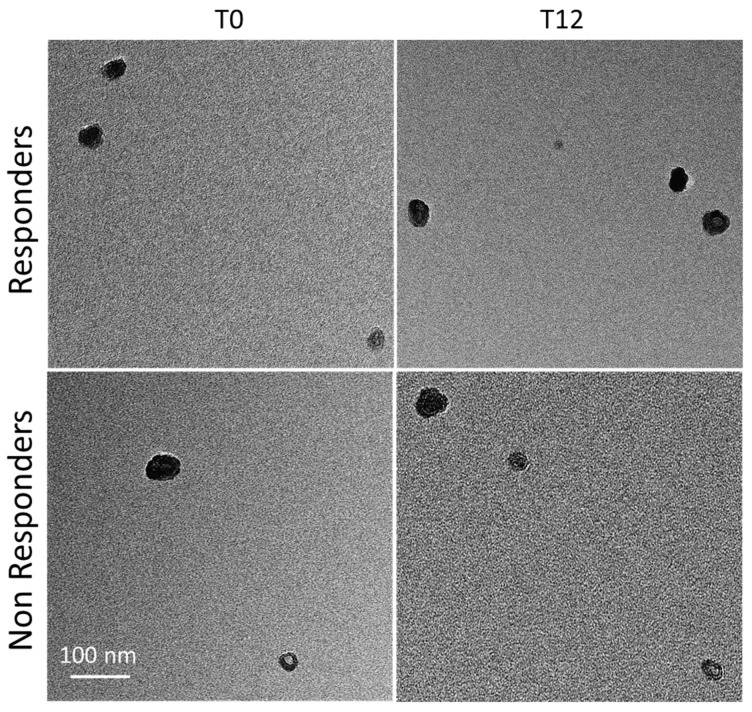
Exosomes extracted from sera of patients with MASLD and T2D, responders and non-responders to semaglutide at baseline (T0) and after 12 months of treatment (T12). Representative TEM micrographs were obtained with staining for 30 s (scale bar 100 nm).

Transmission electron microscopy (TEM) analysis has been employed for morphology studies. It is important to note that when exosomes are cast on the TEM grid for TEM observation, they undergo dehydration, resulting in a loss in volume. Dynamic light scattering (DLS) has been utilized to characterize freshly extracted exosomes in terms of size, size distribution, and surface charge (ζ-potential). In the representative TEM micrographs (Figure 1), distinctive circular structures with a cup-shaped morphology, ascribed to dried exosomes, were observed. DLS analysis, performed on all isolated freshly exosome samples, yielded average hydrodynamic diameters ranging from 134 to 156 nm, while a negative surface charge was recorded by ζ-potential measurement (Table 2).

### 2.3. Membrane Cell Composition (Lipidomic) after Treatment with Exosomes

Lipidomics was carried out to assess the cell membrane composition of HEPA-RG and LX-2 cells treated with T0 and T12 exosomes from R and NR patients. Untreated cells were used as the negative control. The lipidic composition of cell membranes of both cells is reported extensively in Table 3. Membrane fluidity was evaluated by calculating the saturation index (SI; stearic-to-oleic acid ratio). Membrane SI was significantly lower than the LX-2 cells treated with T0 exosomes from R patients (SI: 4.96) compared to untreated cells (** *p* < 0.001) and with both T0 and T12 exosomes from NR patients (SI: 9.01 and 8,39, respectively; * *p* < 0.005 versus untreated cells). Conversely, membrane plasticity was almost restored (SI: 10.87) compared with the controls in LX-2 cells treated with T12 exosomes from R patients (SI: 10.74). No significant differences in membrane SI were observed in HEPA-RG cell lines treated with T0 and T12 exosomes derived from both R and NR patients.

### 2.4. Effect of Exosomes on Liver Cells Viability

HEPA-RG and LX-2 cell lines were treated with T0 and T12 exosomes isolated from R and NR patients for 24 h, 48 h, and 72 h, and cell viability was then quantified using crystal violet and MTS methods.

**Figure 2:**
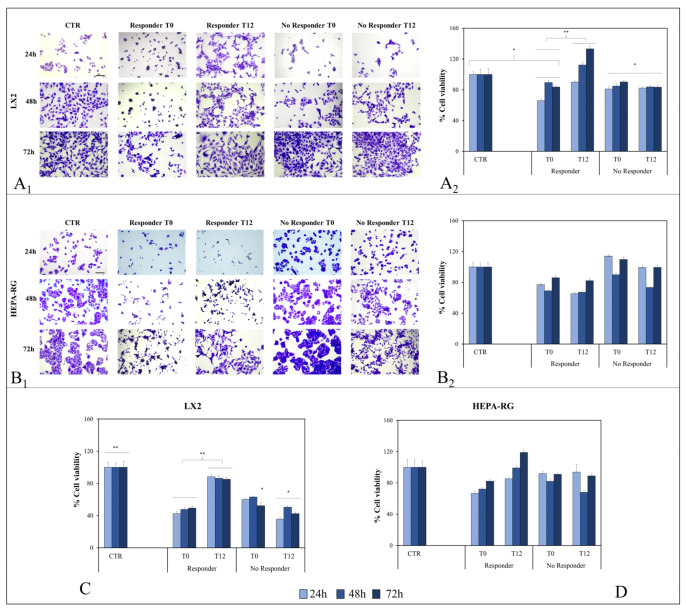
Crystal violet staining micrographs for LX2 (**A_1_**) and Hepa-RG (**B_1_**) detected by a phase-contrast microscope (scale bar 50 μm) and corresponding analysis of total number of cells (**A_2_**,**B_2_**) after cell incubation with exosomes from R and NR patients over the course of 24, 48 and 72 h. Cell viability evaluated by MTS assay of LX2 (**C**) and Hepa-RG (**D**) after cell treatment with exosomes from R and NR patients over the course of 24, 48, and 72 h. ** *p* < 0.001 and * *p* < 0.005.

As shown in Figure 2, a significant reduction (* *p* < 0.005) in LX-2 cell viability was observed when cells were treated with T0 exosomes from R patients, but viability was significantly restored (** *p* < 0.001) when cells were treated with exosomes from R patients after one year of semaglutide therapy. Conversely, when cells were exposed to T0 exosomes derived from NR patients, viability was significantly (** *p* < 0.001) lower than controls and did not change when cells were treated with T12 exosomes from NR patients after 12 months of therapy (Figure 2A_1_,A_2_). To confirm the results obtained, MTS was also performed, which basically confirmed what was observed with the crystal violet method (Figure 2C).

The same experiment was conducted on HEPA-RG cells (Figure 2B_1_,B_2_), but no relevant changes in cell viability were found after cell exposure to T0 and T12 exosomes from both R and NR patients. Furthermore, in the MTS assay, the same data were obtained as with crystal violet (Figure 2D). 

### 2.5. Molecular Characterization of Gene Expression Involved in Liver Fibrosis

The levels of the gene expressions of COL1A1, VIM, α-SMA, and FN1 were evaluated by droplet digital PCR (ddPCR) in untreated cells (CTR), and in LX-2 cells treated with exosomes from R and NR patients at T0 and T12. 

**Figure 3:**
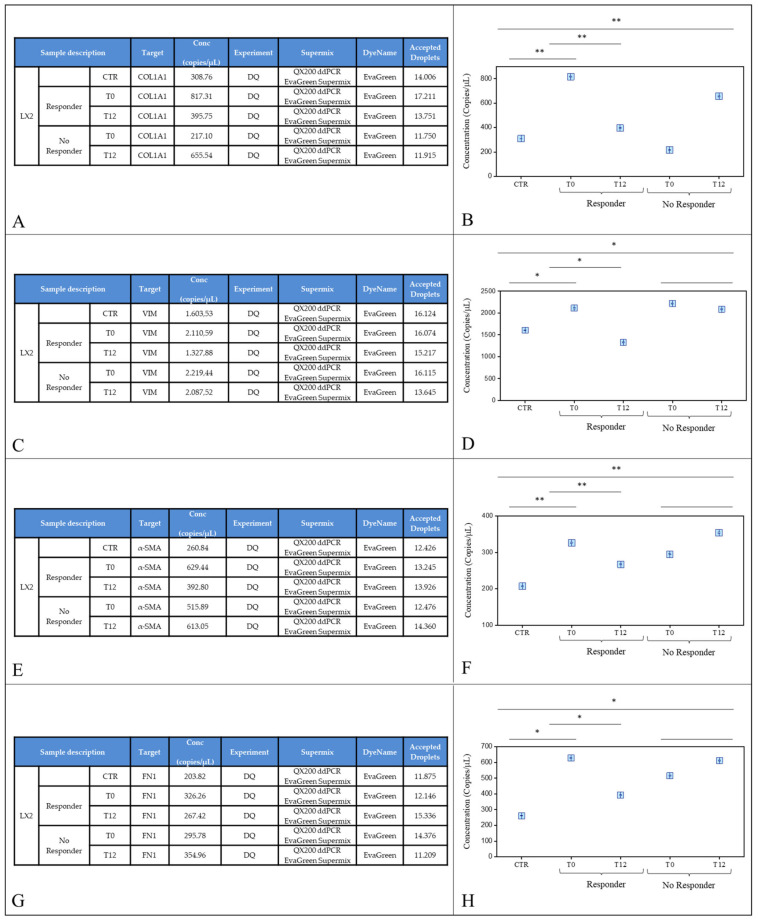
Droplet digital PCR analysis of COL1A1, Vimentin, α-SMA, and Fibronectin. Control LX-2 cells (CTR) and LX-2 cells treated with plasma-derived exosomes from patients with MASLD and T2D and responders and non-responders at T0 (before therapy with semaglutide) and after 12 months of treatment with once-weekly semaglutide 0.5 mg (T12). Value of copies/μL for COL1A1 is reported in (**A**). Average values are reported in (**B**). Value of copies/μL for VIMentin is reported in (**C**). Average values are reported in (**D**). Value of copies/μL for α-SMA is reported in (**E**). Average values are reported in (**F**). Value of copies/μL for Fibronectin is reported in (**G**). Average values are reported in (**H**). *p*-value was determined by one-way ANOVA, * *p* < 0.005 and ** *p* < 0.001.

The expression of COL1A1 in LX-2 cells after 24 h of treatment with exosomes is reported in Figure 3A,B. After a significant increase in COL1A1 expression in cells treated with exosomes from R patients at T0 (** *p* < 0.001) compared with control cells, a significant decrease in gene expression (** *p* < 0.001; Figure 3A) was found when cells were treated with exosomes from responders at T12 compared with cells treated with T0 exosomes from the same group of patients. Conversely, no significant changes were observed with respect to controls when cells were treated with both T0 and T12 exosomes from NR patients (** *p* < 0.001 Figure 3B). The correct number of events and the number of droplets evaluated are reported in Figure S1A of Supplementary Information. Similarly, the mRNA expression levels of VIM in LX-2 cells treated with T0 exosomes from responders were significantly higher than in untreated cells (* *p* < 0.005) but decreased significantly (Figure 3C) after the exposure of LX-2 cells to T12 exosomes from the same group of patients. No changes were recorded in cells treated with T0 and T12 exosomes from NR patients (* *p* < 0.005 Figure 3D). The correct number of events and the number of droplets evaluated are reported in Figure S1B of Supplementary Information. α-SMA is usually poorly expressed by hepatic stellate cells, and its expression is directly related to liver fibrogenesis when these cells differentiate into fibroblasts under pathological conditions. We found a high level of α-SMA expression in cells treated with exosomes from both R and NR patients at T0 compared to control cells (Figure 3E). A significantly lower expression was observed in cells treated with exosomes from R patients at T12, while the α-SMA copy number remained significantly and persistently elevated compared with controls in cells treated with exosomes from NR patients at T12 (** *p* < 0.001, Figure 3F). As with the other genes, the correct number of events and the number of droplets evaluated are reported in Figure S1C of Supplementary Information. This study also looked at FN1, a gene playing a crucial role in liver fibrosis, as it is usually required for collagen matrix assembly and to support other matrix proteins. As shown in Figure 3G,H, we observed a significant increase in FN1 expression in cells treated with exosomes from both R and NR patients at T0 compared with controls (* *p* < 0.005). A significantly lower level of FN1 expression (* *p* < 0.005) was found when cells were treated with exosomes from R patients (T12) compared to untreated cells and when cells were treated with exosomes from both patient groups at T0 (* *p* < 0.005) and with T12 exosomes from NR patients. The correct number of events and the number of droplets evaluated are reported in Figure S1D of Supplementary Information.

### 2.6. Expression of Proteins Involved in Liver Fibrosis in LX-2 Cells Treated with Exosomes

The expression levels of several proteins, namely epidermal growth factor receptor (EGFR), collagen type I alpha 1 chain (COL1A1), matrix metalloproteinase-9 (MMP9), matrix metalloproteinase-2 (MMP2), phosphor-mothers against decapentaplegic homolog 2 (ph-SMAD2), Vimentin (Vim), transforming growth factor-beta1 (TGF-β1), alpha-smooth muscle actin (α-SMA), and connective tissue growth factor (CTGF) involved in the induction and progression of liver fibrosis and in the regulation of signal transduction were analyzed by Western blotting. Analyses were performed on samples extracted from LX-2 cell lines previously treated with exosomes isolated from patients (at T0 and T12). Controls were the protein expression levels in untreated cells.

**Figure 4:**
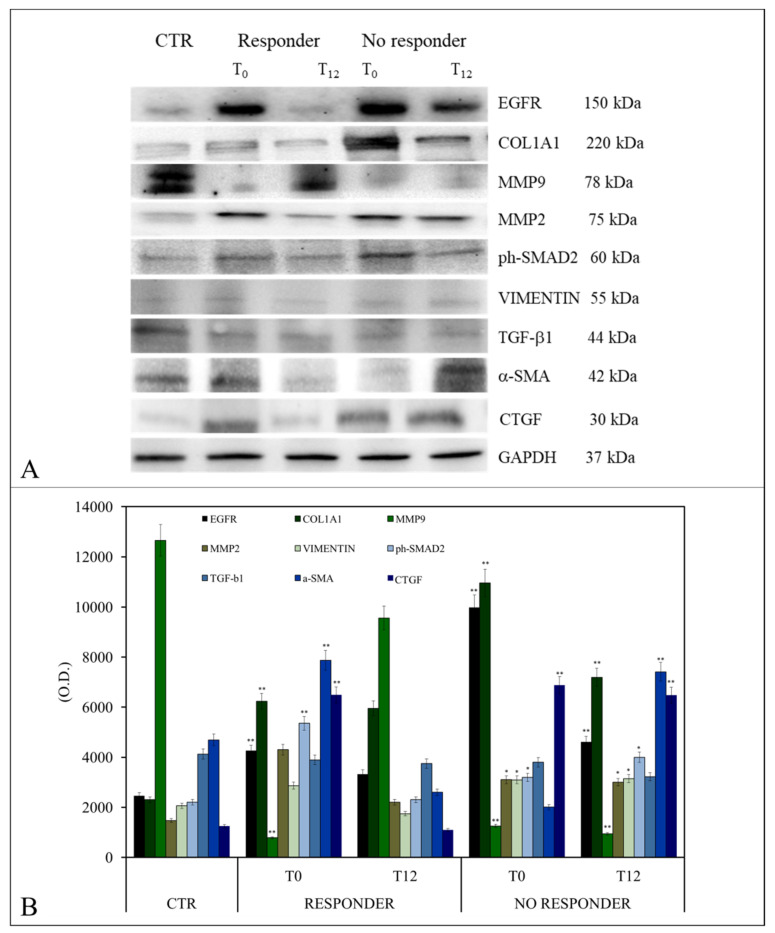
Evaluation of proteins regulating liver fibrosis in LX-2 cells treated with exosomes isolated from patients with MASLD and T2D and responders or non-responders to once-weekly semaglutide 0.5 mg. Representative Western blotting of different proteins (EGFR, COL1A1, MMP9, MMP2, Vimentin, TGF-β1, α-SMA, ph-SMAD2, and CTGF) and housekeeping protein (GAPDH) (**A**). Semiquantitative evaluation of the considered protein expression levels in LX-2 treated cells with exo-somes obtained from patients with T2D and MASLD, both R and NR, by video-densitometry analysis of EGFR, COL1A1, MMP9, MMP2, Vimentin, TGF-β1, α-SMA, ph-SMAD2, and CTGF bands on Western blotting. The GAPDH protein band was used for normalization of the protein band for each subject. (*) *p* < 0.005 and (**) *p* < 0.001 vs. control (CTR) (**B**).

Representative western blotting is shown in Figure 4A. As pictured, cells treated with exosomes from R patients at T0 displayed a significantly higher expression in COL1A1 (** *p* < 0.001), α-SMA (** *p* < 0.001), ph-SMAD2 (** *p* < 0.001), CTGF (** *p* < 0.001), and MMP2 (* *p* < 0.005), while the expression of metalloproteases, namely MMP9 (** *p* < 0.001), was low. No significant changes in Vim and TGF-β1 occurred compared to control cells. 

When cells were treated with exosomes from R patients at T12, all markers were similarly expressed to those observed in controls, except for COL1A1 which was significantly higher (* *p* < 0.005). When cells were treated with exosomes from NR patients, the expression level of MMP9 was lower compared to control cells when either T0 or T12 exosomes were used (** *p* < 0.001). In the case of MMP2, the expression significantly increased when T0 or T12 exosomes were used (* *p* < 0.005) compared with the control group. Moreover, significant increases in Vim (* *p* < 0.005), ph-SMAD2 (* *p* < 0.005), and CTGF (** *p* < 0.001) were observed compared with controls after cell exposure to exosomes from NR at T0 and T12. Finally, a significant increase in α-SMA expression was found in cells treated with T12 exosomes, but not T0 exosomes, from NR patients (Figure 4B). 

**Figure 5:**
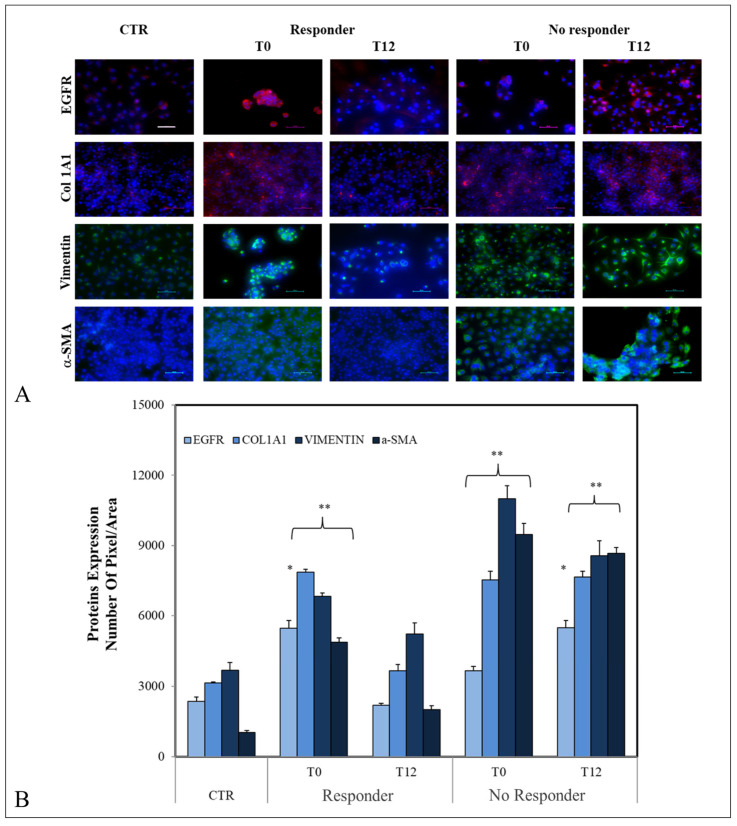
Representative confocal microscopy images of LX-2 cells for the detection of EGFR, COL1A1, Vim, and α-SMA by immunofluorescence. Blue channel: nuclei; red channel: labeled EGFR or COL1A1; green channel: labeled VIM or α-SMA and corresponding overlay. Scale bar: 50 μm. Magnification: ×20. (**A**). Semiquantitative evaluation of EGFR, COL1A1, VIM, and α-SMA expression levels in LX-2 treated with exosomes extracted from patients with MASLD and T2D by fluorescence expression levels, quantitatively evaluated as mean green and red fluorescence intensity index in cells by immunofluorescence. (*) *p* < 0.005 and (**) *p* < 0.001 vs. CTR untreated cells (**B**).

To better understand the effects of exosomes isolated from semaglutide-treated patients, the expressions of EGFR, Col1A1, Vim, and α-SMA were detected by confocal microscopy on LX-2 cells (Figure 5A). Higher expression levels of all these proteins were found in cells treated for 24 h with T0 exosomes from both R and NR patients (* *p* < 0.005 for EGFR in R and ** *p* < 0.001 for the other considered proteins) compared with control cells. The same occurred when LX-2 cells were exposed to the exosomes of NR isolated at T12 (** *p* < 0.001) (Figure 5B). Of note, in cells treated with exosomes from R patients at T12, the expression levels of EGFR, COL1A1, and Vim were comparable to those observed in control cells, while the significant increase in the α-SMA expression level was confirmed.

## 3. Discussion

**Figure 6:**
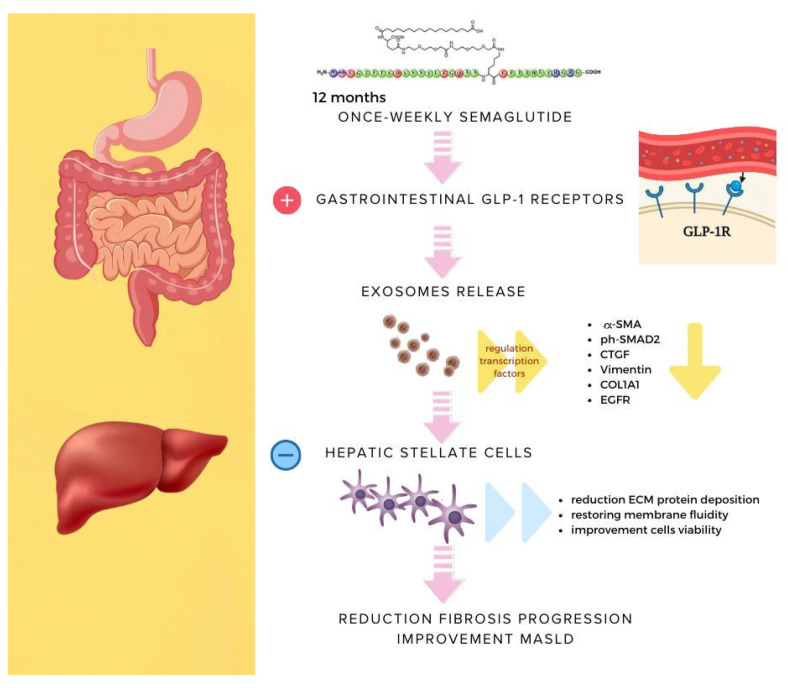
Schematic representation of the possible mechanism mediating the effect of semaglutide in improving/preventing fibrosis progression in MASLD. Exosome release induces a decrease in several proteins involved in ECM deposition (α-SMA, ph SMAD2, CTGF, Vimentin, COL1A1, and EGFR), resulting in restoration of membrane fluidity and improvement of cell viability.

Nowadays, MASLD is the most common metabolic disorder in liver disease. The disease is not only a specific burden on the liver, as the progression from MASLD to MASH and cirrhosis is a well-characterized phenomenon in which stellate cells play a crucial role [18], but it is also known that MASLD increases cardiometabolic risks in dysmetabolic patients. Recent studies in a mouse model suggest that semaglutide normalizes glucose metabolism and reduces body weight, also improving liver steatosis, as demonstrated by reductions in the histological hallmarks of hepatocyte steatosis, such as balloon degeneration and inflammatory foci [19]. It is unclear whether GLP-1RAs, including semaglutide, exert the pharmacological effect by acting directly on specific receptors or by modulating specific intracellular signaling pathways in hepatocytes [20], or indirectly, especially by inducing relevant weight loss [21]. Moreover, it is unclear whether semaglutide may affect the progression of MASLD to hepatic fibrosis and cirrhosis and how GLP-1RAs may affect the activity of stellate cells. Evidence indicates that GLP1-RAs exert an indirect effect on MASLD by improving insulin sensitivity, restoring glucose control, and reducing body weight and adipose tissue, especially at the visceral site [21,22,23]. However, other studies hypothesized that this class of medications may affect lipid metabolism in the liver directly [20,24,25]. In previous studies, the GLP-1 receptor was detected in human hepatocyte cellular lines (HuH7 and HepG2) [25] and in human biopsies of MASH liver, but other studies have not confirmed these data [26,27,28]. The low levels of hepatic GLP-1 receptors detected in some studies could reflect the presence of the GLP-1 receptor in non-hepatocyte cells, such as immune cells, satellite cells, or blood vessels [25,26,29]. In addition, it could derive from a different liver metabolic status, as supposed by other authors who found significant GLP-1 receptor expression in MASH liver and only weak expression in normal liver [24]. In this scenario, we hypothesize that the effectiveness of GLP-1RAs may be attributable to soluble indirect factors downstreaming the activity of the GLP-1 receptors. More precisely, exosomes released after semaglutide treatment by other cells expressing the GLP-1 receptors, such as gastrointestinal cells, may play a crucial role in deactivating LX-2 cells, an in vitro model of liver stellate cells. According to our hypothesis, these soluble particles are the results of the enterocyte exocytosis of the regulators of transcription factors under the effect of the chronic administration of semaglutide (1 year in the experiment described). Exosomes from R patients, those exhibiting a clinical improvement of liver steatosis, were found to decrease α-SMA, COL1A1, Vimentin, and EGFR expressions by LX-2 cells (stellate cells), leading to a decrease in the extracellular matrix (ECM) protein deposition and fibrosis. On the contrary, no results were obtained when LX-2 cells were cultured with exosomes from NR patients, as LX-2 cells maintained the same level of expression of α-SMA as a baseline. Our experiments demonstrated several mechanistic phenomena deserving of specific highlights and explanations. First, chronic exposure to semaglutide (12 consecutive months of treatment) significantly changes the composition of exosomes in R but not in NR patients. Changes in exosome composition, in turn, suggest a relevant change in cell cross-talk even if it is unclear how the change in exosome activity reflects on their composition. LX-2 stellate cell membranes exhibited high stiffness after the cells were cultured to exosomes derived from all patients before starting semaglutide and from NR patients at T12. In contrast, the exposure of LX-2 cells to exosomes extracted from R at T12 restored membrane fluidity compared with control cells. In addition, the cell viability of LX-2 cells was better after the exposure to exosomes from R patients at T12 compared to T0. Other studies demonstrated that modulating the exosome composition may have positive results on several outcomes in MASLD [30,31,32]. Our results could help elucidate the mechanism by which GLP1-RAs exert significant effects on MASLD, indicating potential beneficial effects of semaglutide in the early stages of the metabolic-induced and T2D-associated hepatic injuries (i.e., uncomplicated MASLD). Nevertheless, it is still difficult to determine exactly how these effects are reflected in clinical practice. No data corroborate the hypothesis that the GLP1-RAs may revert clinically established liver fibrosis [33,34], and further studies are needed to better understand how these drugs may have a therapeutic rationale for the treatment of MASH and metabolically related liver fibrosis and cirrhosis [35]. In particular, it is intriguing to consider why some patients even showing clinically relevant improvement in glucose control over time and a relevant body weight loss do not experience significant improvement in MASLD severity assessed by liver US. In conclusion, to our knowledge, this is the first study explaining at least in part the mechanism of semaglutide effectiveness on liver fibrosis, for improving the fibrotic liver disease and/or preventing its worsening (Figure 6).

## 4. Materials and Methods

### 4.1. Ethics 

Enrollment and clinical follow-up of patients were conducted in accordance with the general ethical principles for medical research involving human subjects by the Declaration of Helsinki at the Metabolic Disorders Outpatients Clinic of the Interdisciplinary Department of Medicine, Section of Internal Medicine, University of Bari “Aldo Moro” (Italy). The study protocol was formally approved by the Ethics Committee of the University of Bari (n. 6468 version 2, approved on 14 September 2020). Patients provided written informed consent to participate before study entry.

### 4.2. Examined Patients

In all patients, hepatic steatosis was staged ultrasonographically using the General Electrics Logiq E9 ultrasound machine. More precisely, a validated semiquantitative score based on standardized US characteristics of liver echogenicity was used to stage steatosis as absent (0), mild (1), moderate (2), or severe (3) [36]. In addition, liver steatosis and fibrosis were estimated using clinically validated scores, namely the HSI [37] and the APRI score [38], which can be easily calculated from laboratory data. More precisely, the HSI was calculated according to the following formula: 8 × (ALT/AST) + BMI + 2 (if T2D) + 2 (if female). A value above 36 is suggestive of hepatic steatosis, and a value lower than 30 should exclude the disease. The APRI score was calculated using the following formula: AST/AST (upper limit of the reference range)/platelets × 100. Values above 0.5 or 1 were considered indicative of an increased risk of liver fibrosis or cirrhosis, respectively. 

To improve the accuracy of the estimation of liver fibrosis severity, we also calculated FIB-4 [39]. FIB-4 was obtained by the following calculation: (Age × AST)/(PLT × √ALT). A cut-off value < 1.45 has a negative predictive value of 90% for ruling out extensive fibrosis. A cut-off value > 3.25 has a positive predictive value of 65% for the diagnosis of extensive fibrosis.

Sera from 10 consecutive unselected outpatients were obtained before (T0) and after one year (T12) of treatment. Venous samplings were collected in the morning after overnight fasting; specimens were then stored and processed for additional analyses. At the end of follow-up, 6 patients were classified as R and 4 as NR with reference to improvements in liver steatosis, while 8/10 patients had a BMI reduction by more than 5% from baseline.

### 4.3. Cell Culture

The human HEPA-RG hepatoma cell line (Thermo Fisher Scientific, Waltham, MA, USA) was cultured using a hepatocyte bullet kit medium added with 10% FBS, depleted of exosomes (Lonza Biowhittaker, Oslo, Norway), and with 1% of 100 mM Antibiotic-Antimycotic (penicillin 10,000 91 u/mL, streptomycin 10,000 u/mL, (Lonza Biowhittaker, Oslo, Norway). The LX2 Human Hepatic Stellate cell line (Millipore, Merck Life Science, Milan, Italy) was cultured using Dulbecco’s Modified Eagle Medium (DMEM) (Thermo Fisher Scientific, Waltham, MA, USA), added with 10% of FBS depleted exosomes, 1% of 100 M Antibiotic-Antimycotic, 2.5% of HEPES 1M (N-2-hydroxyethylpiperazine-N-2-ethane sulfonic acid) (Gibco™, ThermoFisher Scientific, Waltham, MA, USA) and 1% of 100 mM Sodium Pyruvate (Gibco™, ThermoFisher Scientific, Waltham, MA, USA). For all treatments with exosomes, both cell lines were cultured on semi-confluent cell monolayers and were treated with exosomes derived from patients at a concentration of µg exosome proteins/µL of medium, specifically at 20 µg/µL for 24, 48, and 72 h.

### 4.4. Exosome Extraction and Characterization

The exosomes were extracted from patient sera, using the sequence of centrifugation and ultracentrifugation described in a previous setting protocol [40]. An aliquot was used immediately for exosome characterization; then, the other part was stored at −80 °C until the protein analysis was carried out. The characterization of exosomes was performed using the Jeol Jem-1011 (JEOL USA, Inc, Peabody, MA, USA), a transmission electron microscope working at an accelerating voltage of 100 kV, and the Olympus Quemesa Camera (11 Mpx) (Olympus, Shinjuku-ku, Tokyo 163-0914, Japan) to acquire proper images. The staining of samples was performed according to the experimental procedure reported in a previous paper [41,42]. The hydrodynamic diameter, ζ-potential, and stability with the corresponding polydispersity index (PDI) were investigated with the Zetasizer Nano ZS (Malvern Instruments Ltd., Worcestershire, UK), a dynamic light scattering (DLS) system. The operative instrument condition was a 4 mW He-Ne laser as a light source (wavelength λ = 633 nm). A disposable folded capillary cell, DTS1070 (Malvern Instruments Ltd., Worcestershire, UK) was used, as already reported by Depalo et al. [43]. Three consecutive measurements were performed on each sample to obtain data, reported as average value ± standard deviation.

### 4.5. Fatty Acid Extraction, Purification, and Preparation of Fatty Acid Methyl Esters 

Methanol, chloroform, n-hexane, toluene, boron trifluoride in methanol ≥ 99.5%, hydrochloric acid ACS reagent 37%, sodium sulfate, and sodium chloride were from Sigma-Aldrich (Milan, Italy). The Ficoll-Paque PLUS reagent was obtained from GE Healthcare (Uppsala, Sweden). Larodan 37 FAME Mix (Larodan Fine Chemicals, 90-1100 Mixture ME 100, Monroe MC, USA), methyl palmitelaidate (9,E) (Larodan Fine Chemicals Monroe MC, USA), methyl vaccinate (11,Z) (Larodan Fine Chemicals Monroe MC, USA), methyl hexadecenoate 7(Z) (Larodan Fine Chemicals Monroe MC, USA), and methyl docosapentanoate (7Z,10Z,13Z,16Z,19Z) (Larodan Fine Chemicals Monroe MC, USA) were provided by LABSERVICE S.R.L. (Rome, Italy). The fatty acid methyl ester isomer mix was prepared by dissolving fatty acids methyl esters in n-hexane, stored at −20 °C, and used for qualitative and quantitative analyses. 

The Moilanen method [44], a modified method derived from the Folch method [45], was used for fatty acid extraction from HEPA-RG and LX-2 untreated cell lines. These cell lines were used as negative controls or treated with exosomes isolated from R and NR patients at T0 and T12. Each sample of HEPA-RG and LX-2 cell lines was thawed to room temperature. Fatty acids were hydrolyzed from phospholipids of HEPA-RG and LX-2 membranes by adding 1 mL of acidified salt solution (H_2_SO_4_ 2 × 10^–4^ M, NaCl 0.1%) and were extracted by centrifugation at 1500× *g* for 10 min with chloroform 3 mL and methanol 1.5 mL, following Coviello’s protocol [46]. Extracted fatty acids were analyzed and quantified by gas chromatography using a Thermo-Scientific instrument with auto-sampler, a split/splitless injector, FID detector, and hydrogen gas generator (Thermo Fisher Scientific, Milan, Italy) [46].

### 4.6. Cell Viability Assay 

Hepatic cell lines, namely LX-2 and HEPA-RG, were seeded into 96-well plates at a density of 2 × 10^3^ cells per well and, after 24 h, with exosomes from patients’ sera at 37 °C for 24, 48, and 72 h. Controls were untreated cells. Briefly, both cell lines were seeded and treated in the same way as for the MTS assay (Promega, WI, USA). After the treatment with exosomes, cells in DMEM were fixed with paraformaldehyde (PFA) 4% (Sigma-Aldrich, Milan, Italy) and incubated for 30 min. The DMEM added with PFA 4% was then discarded, and crystal violet was added and incubated for 10 min. The plate was washed with cold water, and before solubilization, images were acquired with the Nikon confocal microscope Eclipse Ti2 in bright field using 20× magnification. Following SDS 1% (Sigma-Aldrich, Milan, Italy), plates were washed again and incubated for 30 min at room temperature. The absorbance was measured at 595 nm (PerkinElmer Victor Plate Reader, Lier Belgium). To corroborate the results obtained with the crystal violet assay, the MTS assay was performed. Briefly, MTS tetrazolium compound reagent (20 μL) for a total volume of 120 μL was added to cells treated in the same way as the cells used to perform the crystal violet assay, and they were incubated at 37 °C for 3 h. The absorbance was captured at 490 nm (PerkinElmer Victor Plate Reader, Lier Belgium). The percentage of cell viability was calculated after the subtraction of absorbance of blank (obtained from the wells without cells) from absorbance of all wells with the cells. Then, the percentage of cell viability was calculated using the following equation:

% Viability = Mean OD samples/Mean OD ctr × 100

### 4.7. RNA Extraction, cDNA Synthesis, and Digital Droplet PCR Analysis

Ribonucleic acid (RNA) extraction was conducted from frozen cell pellets using the RNeasy^®^ Mini kit (QIAGEN, Hilden, Germany). All samples were stored at −80 °C before reverse transcription. The RNA concentration was measured using a NanoDrop Lite (Thermo Fisher Scientific, Waltham, MA, USA). An aliquot of 800 ng of total RNA was transcribed using the iScript™ cDNA Synthesis kit (Bio-Rad, Hercules, CA, USA), following the reaction protocol: 5-min priming at 25 °C; 20-min reverse transcription at 46 °C; one-minute reverse transcription inactivation at 95 °C; holding at 4 °C. Storage was at a temperature of −20 °C. The cDNA was analyzed in the treated HEPA-RG cell line and the LX-2 cell line to evaluate the copy numbers per microliter of VIM, COL1A1, and HGF, quantified by droplet digital PCR (ddPCR; QX200 Droplet DigitalPCR System, Bio-Rad, Hercules, CA, USA) following the manufacturer’s instructions for the EvaGreen protocol. The reaction was conducted with a total volume of 20 μL, including 15 ng of cDNA per sample, except for VIM, for which 5 ng of cDNA per sample were loaded. We used 10 μL of QX200™ ddPCR™ EvaGreen Supermix (Bio-Rad, Hercules, CA, USA), RNase-/DNase-free water (variable), and 100 nM primer SYBR^®^ Green Assay for ddPCR. The primers were purchased from Bio-Rad with the following assay ID numbers: VIM, qHsaCID0012604; col1a1, qHsaCED0002181; α-SMA, qHSACID0013300; FN1, qHSACED0043611; EGFR, qHSACED0045334. Cycling conditions were the following: 1 cycle at 95 °C for 5 min; 40 cycles at 95 °C for 30 s; 40 cycles at 60 °C for 1 min; 1 cycle at 4 °C for 5 min; 1 cycle at 90 °C for 5 min; holding at 4 °C. Data were processed using the QX Manager 1.2 Standard Edition (Bio-Rad, Hercules, CA, USA).

### 4.8. Western Blotting Analysis

Western blotting analysis was performed to evaluate the expression of several proteins, including epithelial growth factor (EGFR), Collagen IA1 (COL1A1), matrix metalloproteinase 2 (MMP2), and 9 (MMP9), vimentin (Vim), alpha-smooth muscle actin (α-SMA), CTGF, ph-SMAD2, and transforming growth factor (TGF)-β1, in LX-2 and HF cell lines after the exposure to exosomes. Cells were treated for 48 h; then, proteins were extracted by the Ripa buffer, supplemented with protease and phosphatase inhibitors (Thermo Scientific, Rockford, IL, USA). 

Protein concentrations were measured by a standard Bradford assay (Bio-Rad, Milan, Italy), and aliquots of 20 µg of total protein extracts were loaded onto 4–15% precast polyacrylamide gels (Bio-Rad Laboratories, Milan, Italy). The gel was blotted onto a polyvinylidene fluoride (PVDF) membrane (Bio-Rad Laboratories, Milan, Italy), and proteins were detected with the following primary antibodies: anti-EGFR, anti-COL1A1, anti-MMP2 and anti-MMP2, anti-Vim, anti-CTGF and anti-ph-SMAD2 (1:500 Cell Signaling Technology, Beverly, MA, USA), anti-α-SMA (1:400 Thermo Fisher Scientific, Waltham, MA, USA), anti-TGFβ1, and anti-glyceraldehyde-3-phosphate-dehydrogenase, (GAPDH; 1:1000 Santa Cruz, CA, USA). After overnight incubation, the membranes were washed and further incubated with the respective secondary antibody. 

Proteins were then detected by enhanced chemiluminescence (ECL, Thermo Fisher Scientific, Waltham, MA, USA). The protein signals were obtained using the Chemidoc Molecular Imager (Bio-Rad, Milan, Italy), and normalized against the GAPDH expression. The images were analyzed using Image Lab 5.2.1, software 4.6. 

### 4.9. Immunofluorescence 

LX-2 cells were seeded into 48 well plates at a density of 5 × 10^3^ cells per well. Cells were treated at 37 °C with exosomes for 24 h, and untreated cells were used as control. Treated and untreated cells were washed with phosphate-buffered saline (PBS), (Thermo Fisher Scientific, Waltham, MA, USA) fixed with 4% paraformaldehyde for 25 min at −20 °C, and permeabilized with Triton X-100 (0.5%) in PBS for 15 min. Then, cells were blocked with normal serum (5%) in PBS for one hour and incubated at 4 °C overnight with the two primary antibodies. A previously described protocol was applied 43 with the following antibodies: anti-EGFR, anti-COL1A1, anti-Vim, (1:200 Cell Signaling Technology, Beverly, MA, USA), and anti-α−SMA (1:200 Thermo Fisher Scientific, Waltham, MA, USA). After washing, the cells were incubated with specific green fluorescent (goat anti-rabbit IgG (H + L) secondary antibody Alexa Fluor 488 conjugate or antibody Alexa Fluor 555 conjugate) secondary antibodies for one hour, (Thermo Fisher Scientific, Waltham, MA, USA) at room temperature, in the dark. Subsequently, the cells were mounted using a vectashield antifade mounting medium with DAPI (Vector Laboratories, Inc. Newark, CA, USA). 

### 4.10. Statistical Analysis

ANOVA was conducted to assess differences in all experiments between control cells and cells treated with exosomes derived from T0 and T12. Additionally, comparisons were made between cells treated with T0 and T12 exosomes derived from T2D patients, both responders and non-responders to therapy with semaglutide. Control cells were consistently treated as the reference category in all analyses. Statistical significance was defined at * *p* ≤ 0.005 and ** *p* ≤ 0.001. Post hoc analysis for multiple comparisons was carried out using the Bonferroni test. The statistical package employed for all analyses was SigmaStat version 3.1. 

## Figures and Tables

**Table 1:** Characteristics of examined patients.

Age (yrs)	Gender	Response Masld/BMI	T2D Evolution (yrs)	BMI T0 [T12] Δ%	HbA1c T0 [T12]	APRI T0 [T12]	HIS T0 [T12]	FIB-4 T0 [T12]	US Grading (0–3) T0 [T12]
59	M	R/R	7	33.5 [30.1] −10%	41 [40]	0.31 [0.37]	40.0 [36.9]	0.94 [1.17]	2 (1)
49	M	R/R	0	39.2 [26.5] −32.4%	41 [40]	0.27 [0.24]	45.7 [33.8]	0.74 [0.84]	3 (0)
54	W	NR/R	1	43.6 [38.3] −12.1%	34 [31]	0.37 [0.15]	52.8 [48.4]	0.98 [0.66]	2 (2)
71	M	R/R	20	37.7 [33.5] −11.1%	49 [49]	0.32 [0.18]	50.4 [41.3]	**2.01** [1.12]	2 (1)
61	W	NR/NR	14	36.5 [34.9] −4.4%	36 [33]	0.25 [0.19]	47.8 [50.5]	1.16 [1.01]	2 (2)
61	W	NR/R	7	35.4 [29.4] −16.9%	38 [36]	0.43 [0.19]	44.7 [40.5]	1.33 [1.08]	2 (2)
42	W	R/R	0	41.6 [35.7] −14.2%	38 [38]	0.16 [0.11]	52.3 [43.8]	0.49 [0.34]	3 (2)
62	W	R/R	2	37.5 [31.2] −16.8%	43 [37]	0.17 [0.24]	92.1 [53.6]	**2.25** [1.76]	2 (1)
47	M	R/NR	1	35.6 [34.1] −4.2%	34 [38]	0.25 [0.11]	43.7 [39.4]	0.67 [0.34]	3 (2)
60	M	NR/R	1	32.4 [29.1] −10.2%	73 [53]	0.23 [0.15]	38.9 [37.1]	0.81 [0.76]	2 (2)

Abbreviations: M = man; W = woman; R = responder; NR = non-responder; MASLD = metabolic dysfunction-associated steatotic liver disease; T2D = type 2 diabetes; BMI = body mass index (kg/m^2^); Δ: change from T0 (%); HbA1c = glycated hemoglobin (mmol/mol); APRI = aspartate aminotransferase to platelet ratio index; HIS = hepatic steatosis index; FIB-4 = fibrosis-4 index for liver fibrosis; US = ultrasonographic.

**Table 2:** The intensity average hydrodynamic diameters (size nm) of the exosome cargo, polydispersity indexes (PDI), and ζ-potential values.

Sample	Size (nm)	PDI	ζ-Potential (mV)
Responders T0	136 ± 3	0.258 ± 0.012	−29.1 ± 0.1
Responders T12	131 ± 2	0.239 ± 0.006	−25.5 ± 0.1
Non-Responders T0	156 ± 4	0.266 ± 0.004	−26.5 ± 0.3
Non-Responders T12	154 ± 2	0.226 ± 0.011	−27.6 ± 0.4

**Table 3:** Membrane cell composition in HEPA-RG and LX-2 cells. The stearic-to-oleic acid ratio (Saturation index: SI) worsened after LX-2 cells were exposed to exosomes from R and NR patients before starting semaglutide (T0) and from NR patients after 12 months of therapy (T12), while it improved in LX-2 cells exposed to exosomes from R patients at T12. No specific changes were observed in HEPA-RG cells. ** *p* < 0.001 and * *p* < 0.005. For the blue cells, a significant variation in the saturation index in LX2 cell line after treatment with exosomes.

Fatty Acids (%)	HEPA-RG Cells						LX2 Cells					
			Responders		No Responders				Responders		No Responders	
	CTR	Healthy	T_0_	T_12_	T_0_	T_12_	CTR	Healthy	T_0_	T_12_	T_0_	T_12_
**Palmitic acid (C16:0)**	34.15	40.30	46.20	45.51	43.31	42.52	44.44	48.91	41.14	42.43	40.88	40.37
**Palmitoleic acid (C16:1n7)**	2.24	0.80	2.06	2.18	2.04	1.84	1.25	0.83	1.60	1.50	1.58	2.04
**7z-palmitoleic acid (C16:1n9)**	5.27	0.82	2.36	2.93	2.61	2.53	0.49	0.00	0.65	0.49	0.65	1.06
**Stearic acid (C18:0)**	19.78	33.94	28.81	27.74	27.48	27.14	21.96	31.96	26.91	24.67	29.06	28.67
**Elaidic acid (C18:1trans)**	21.39	10.36	14.34	15.07	18.21	18.72	14.57	3.09	14.15	19.80	20.30	19.43
**C18:1n9**	11.70	1.03	3.62	4.51	4.91	5.25	2.21	4.06	3.63	2.84	3.24	3.44
**Arachidonic acid (C20:4n6)**	2.57	2.26	2.40	2.38	1.44	1.89	2.85	1.15	4.07	3.52	3.56	4.20
**Saturation index** **(C18:0/C18:1n9)**	1.69	32.84	10.34	9.59	5.82	5.18	15.20	10.87	4.96 **	10.74	9.01 *	8.39 *
**∆9-desaturase (C18:1n9/C18:0)**	0.59	0.03	0.11	0.13	0.18	0.19	0.07	0.13	0.15	0.08	0.11	0.12
**∆9-desaturase (C16:1n7/C16:0)**	0.07	0.02	0.05	0.05	0.05	0.04	0.01	0.00	0.01	0.01	0.02	0.03
**Elovl6 (C18:0/C16:0)**	0.58	0.67	0.62	0.63	0.63	0.64	0.49	0.54	0.71	0.58	0.71	0.71
**β-oxidation (C16:1n9/C18:1n9)**	0.45	0.79	0.64	0.94	0.54	0.48	0.22	0.00	0.19	0.19	0.21	0.29

## Data Availability

The original contributions presented in the study are included in the article/supplementary material, further inquiries can be directed to the corresponding author/s.

## Supplementary Materials

The supporting information can be downloaded at: https://www.mdpi.com/article/10.3390/ijms25031493/s1.
