# Supplementary material for: Semaglutide Modulates Extracellular Matrix Production of LX-2 Cells via Exosomes and Improves Metabolic Dysfunction-Associated Steatotic Liver Disease (MASLD)
Source: Int J Mol Sci. 2024 Jan 25;25(3):1493. doi: 10.3390/ijms25031493 (PMC10855465; doi:10.3390/ijms25031493)
Supplement: Supplementary file 1 [file ijms-25-01493-s001.zip › ijms-2805482-supplementary.docx]

**SUPPORTING INFORMATION**

**Semaglutide Improves Metabolic Dysfunction Associated Steatotic Liver Disease (MASLD) Modulating Extracellular Matrix Production of LX-2 cells via Exosomes**

*Maria Principia Scavo^1*^, Giuseppe Lisco^2^ , Nicoletta Depalo^3^, Federica Rizzi^3^, Sara Volpe^2^, Valentina Arrè^1^, Livianna Carrieri ^1^, Maria Notarnicola^4^, Valentina De Nunzio^4^, Maria Lucia Curri^5^, Giovanni De Pergola^6^, Giuseppina Piazzolla^2^* *^ѯ^ and Gianluigi Giannell^7^* *^ѯ^*

1. Laboratory of Personalized Medicine, National Institute of Gastroenterology, IRCCS DeBellis, Castellana Grotte (BA), Italy. [maria.scavo@irccsdebellis.it](mailto:maria.scavo@irccsdebellis.it); [valentina.arre@irccsdebellis.it](mailto:valentina.arre@irccsdebellis.it); [livianna.carrieri@irccsdebellis.it](mailto:livianna.carrieri@irccsdebellis.it);
2. Interdisciplinary Department of Medicine, School of Medicine, University of Bari “Aldo Moro”, Piazza Giulio Cesare 11, 70124 Bari, Italy. [giuseppe.lisco@uniba.it](mailto:giuseppe.lisco@uniba.it); [svolpe.doc@gmail.com](mailto:svolpe.doc@gmail.com) ; [giuseppina.piazzolla@uniba.it](mailto:giuseppina.piazzolla@uniba.it)
3. Institute for Chemical-Physical Processes, Italian National Research Council (IPCF)-CNR SS Bari, Via Orabona 4, 70125 Bari, Italy. [n.depalo@ba.ipcf.cnr.it](mailto:n.depalo@ba.ipcf.cnr.it); f.rizzi@ba.ipcf.cnr.it
4. Laboratory of Nutritional Biochemistry, National Institute of Gastroenterology "S. de Bellis" Research Hospital, 70013 Castellana Grotte Bari, Italy. [maria.notarnicola@irccsdebellis.it](mailto:maria.notarnicola@irccsdebellis.it); [valentina.dinunzio@irccsdebellis.it](mailto:valentina.dinunzio@irccsdebellis.it);
5. Dipartimento di Chimica, Università degli Studi di Bari Aldo Moro, Via Orabona 4, 70125 Bari, Italy. marialucia.curri@uniba.it
6. Center of Nutrition for the Research and the Care of Obesity and Metabolic Diseases, National Institute of Gastroenterology IRCCS "Saverio de Bellis", 70013 Castellana Grotte, Italy. [giovanni.depergola@irccsdebellis.it](mailto:giovanni.depergola@irccsdebellis.it).
7. Scientific Direction, National Institute of Gastroenterology IRCCS "De Bellis," Via Turi 27 Castellana Grotte, Bari, Italy. [gianluigi.giannelli@irccsdebellis.it](mailto:gianluigi.giannelli@irccsdebellis.it)

*^*^*Correspondence: Maria Principia Scavo maria.scavo@irccsdebellis.it (M.P.S.) phone: +39-080-4994697;

*^ѯ^* These authors contributed equally to this work: Giuseppina Piazzolla giuseppina.piazzolla@uniba.it (G.P.) and Gianluigi Giannelli [gianluigi.giannelli@irccsdebellis.it](mailto:gianluigi.giannelli@irccsdebellis.it) (GG)

**Droplet digital PCR analysis of COL1A1, Vimentin, α-SMA, and Fibronectin.**

The levels of gene expression of COL1A1, VIM, α-SMA, and FN1, were evaluated by the droplet digital PCR (ddPCR) in untreated cells (CTR), and in LX-2 cells treated with exosomes from R and NR patients at T0 and T12. Herein the correct number of events and the number of droplets evaluated are reported in figure 1S for each gene expression, in particular in figure 1SA are reported the number of droplets and events generated for Collagene 1A1, in figure 1BS, are reported the number of droplets and events generated and analyzed for Vimentin gene. In the figure 1CS are reported the number of droplets and events for a-SMA, while in figure 1DS we reported the number of droplets and events for fibronectin. All of this experiments for all gene considerated suggest the correct number of droplets, more than 10000 and the number of event higher more than 10000.


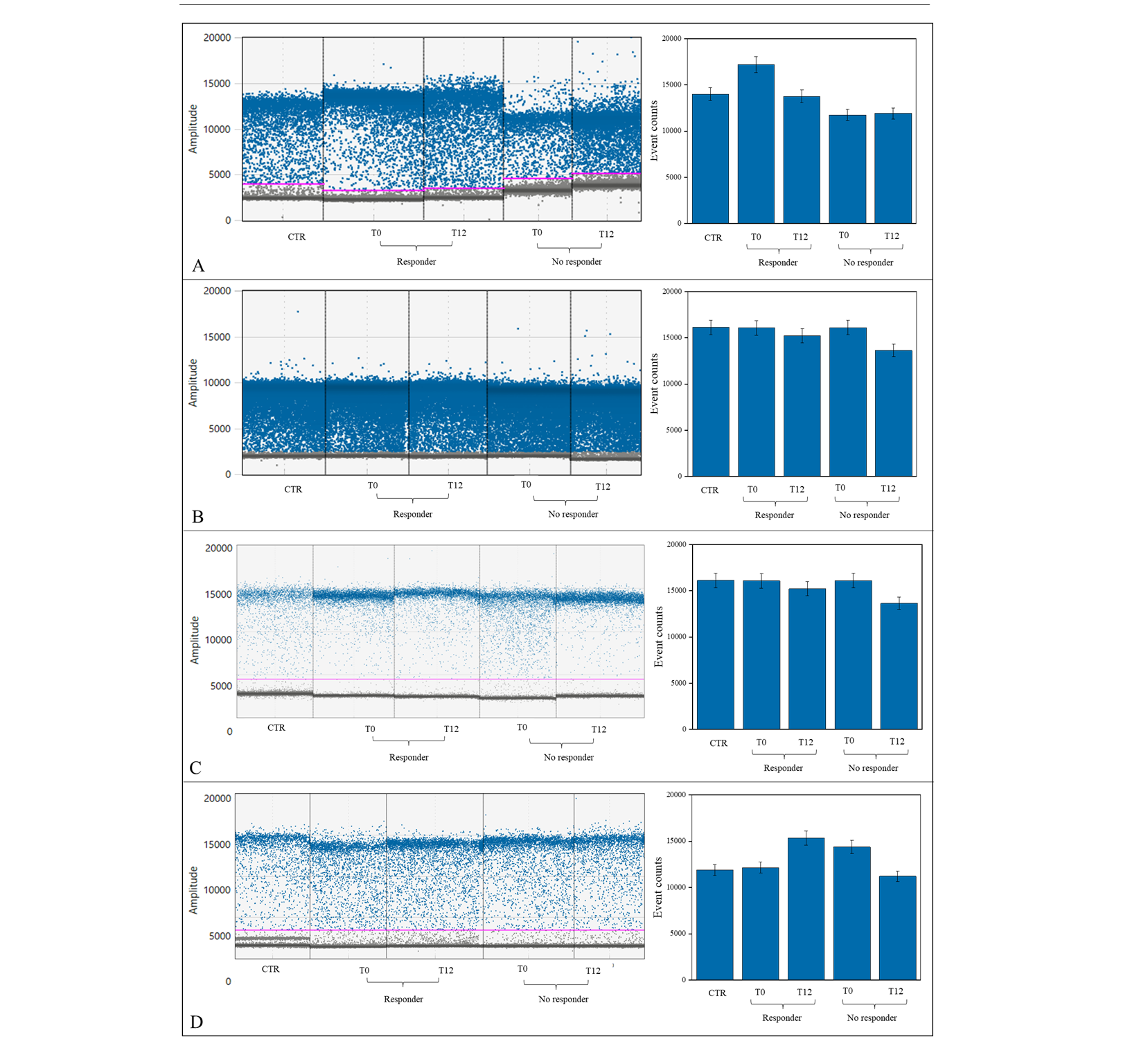


**Figure S1. Droplet digital PCR analysis of COL1A1, Vimentin, α-SMA, and Fibronectin.** Control LX-2 cells (CTR), LX-2 cells treated with plasma-derived exosomes from patients with MASLD and T2D, Responders and Non-Responders at T0 (before therapy with semaglutide) and after 12 months of treatment with once-weekly semaglutide 0.5 mg (T12). Number of droplets and events for COL1A1 were reported in panel A. Number of droplets and events for Vimentin were reported in panel B. Number of droplets and events for α-SMA were reported in panel C. Number of droplets and events for Fibronectin were reported in panel D.
